# Luteal Lipids Regulate Progesterone Production and May Modulate Immune Cell Function During the Estrous Cycle and Pregnancy

**DOI:** 10.3389/fendo.2019.00662

**Published:** 2019-10-04

**Authors:** Camilla H. K. Hughes, Remy Bosviel, John W. Newman, Joy L. Pate

**Affiliations:** ^1^Center for Reproductive Biology and Health, Department of Animal Sciences, Pennsylvania State University, State College, PA, United States; ^2^West Coast Metabolomics Center, Genome Center, University of California, Davis, Davis, CA, United States; ^3^Obesity and Metabolism Research Unit, USDA-ARS-Western Human Nutrition Research Center, Davis, CA, United States; ^4^Department of Nutrition, University of California, Davis, Davis, CA, United States

**Keywords:** corpus luteum, metabolomics, lipoxygenase, immune cells, progesterone, pregnancy, cell proliferation

## Abstract

Although the corpus luteum (CL) contains high concentrations of lipid in the form of steroid hormone precursors and prostaglandins, little is known about the abundance or function of other luteal lipid mediators. To address this, 79 lipid mediators were measured in bovine CL, using ultra performance liquid chromatography-tandem mass spectrometry. CL from estrous cycle days 4, 11, and 18 were compared and, separately, CL from days 18 of the estrous cycle and pregnancy were compared. Twenty-three lipids increased as the estrous cycle progressed (*P* < 0.05), with nine increasing between days 4 and 11 and fourteen increasing between days 4 and 18. Overall, this indicated a general upregulation of lipid mediator synthesis as the estrous cycle progressed, including increases in oxylipins and endocannabinoids. Only 15-KETE was less abundant in the CL of early pregnancy (*P* < 0.05), with a tendency (*P* < 0.10) for four others to be less abundant. Notably, 15-KETE also increased between estrous cycle days 4 and 18. Ingenuity Pathway Analysis (IPA, Qiagen) indicated that functions associated with differentially abundant lipids during the estrous cycle included leukocyte activation, cell migration, and cell proliferation. To investigate changes in CL during maternal recognition of pregnancy, this lipid dataset was integrated with a published dataset from mRNA profiling during maternal recognition of pregnancy. This analysis indicated that lipids and mRNA that changed during maternal recognition of pregnancy may regulate some of the same functions, including immune cell chemotaxis and cell-cell communication. To assess effects of these lipid mediators, luteal cells were cultured with 5-KETE or 15-KETE. One ng/mL 5-KETE reduced luteal progesterone on day 1 of culture, only in the absence of luteinizing hormone (LH), while 1 ng/mL 15-KETE induced progesterone only in the presence of LH (10 ng/mL). On day 7 of culture, 0.1 ng/mL 15-KETE reduced prostaglandin (PG)F2A-induced inhibition of LH-stimulated progesterone production, while 1 ng/mL 15-KETE did not have this effect. Overall, these data suggest a role for lipid mediators during luteal development and early pregnancy, as regulators of steroidogenesis, immune cell activation and function, intracellular signaling, and cell survival and death.

## Introduction

The corpus luteum (CL) originates from the ovulatory follicle and has an incredibly high steroidogenic output. Its primary steroid hormone product is progesterone, which is produced from cholesterol precursor and is the hormone that maintains pregnancy in all mammals, making the CL necessary to normal reproductive function. In the absence of a viable pregnancy, the bovine CL regresses rapidly, in response to prostaglandin (PG) F2A, ceasing progesterone production and allowing another ovulation to occur. Therefore, luteal rescue in the presence of a pregnancy is also imperative for reproduction. Many luteal functions and processes are conserved; therefore, an improved understanding of luteal function in the cow may be applied to our understanding women's health.

The CL is a site of bioactive lipid synthesis. It contains high concentrations of arachidonic acid (1) and luteal tissue uptake of arachidonic acid *in vitro* was greater than the uptake of this prostaglandin precursor in skeletal muscle or ovarian cortex (2). Free arachidonic acid was present in greater concentrations in the CL of the estrous cycle than in the CL of pregnancy on day 18 (1), perhaps providing precursor for the luteal synthesis of PGF2A that is required for luteal regression (3). Intraluteal synthesis of PGEs and prostacyclin and uterine synthesis of PGEs have both been implicated in luteal rescue during early pregnancy (4–7), although this mechanism is not entirely clear.

Although prostaglandin products of arachidonic acid have been studied extensively in the CL, functions of other intraluteal lipids are less well-understood. Arachidonate lipoxygenases may act upon arachidonic acid to form hydroxyeicosatetraenoic acids, lipoxins, and leukotrienes. Uterine infusion of a lipoxygenase inhibitor delayed return to estrous, implicating lipoxins in regulation of estrous cycle length (8) and perhaps in PGF2A synthesis and luteal function. Specific 5-lipoxygenase (ALOX5) products including lipoxins and leukotrienes also altered luteal function in *in vitro* assays. 5-HETE and leukotriene (LT) C4 both reduced progesterone production and 5-HETE also reduced prostacyclin, while LTC4 induced PGF2A. Conversely, LTB4 induced progesterone and PGE2 production in cultured luteal cells (8, 9). These data indicate that leukotrienes and lipoxins may be luteotropic or luteolytic, depending on the molecule, yet little is known about intraluteal concentrations of these lipids.

Fatty acid-derived mediators from other polyunsaturated fatty acids may also have important ovarian functions. Surprisingly, supplementation of fatty acids in the form of flaxseed to transition cows increased the diameter of the corpus luteum (10) and fatty acid supplementation to cows in the form of fish meal resulted in altered omega-3 fatty acid concentrations in plasma and decreased luteal sensitivity to PGF2A, perhaps through a mechanism involving lateral mobility of the prostaglandin F receptor in the cell membrane (11, 12). These data indicate that dietary fatty acid content may alter luteal function.

Despite data indicating an important functional role for bioactive lipids in luteal function, little is known about the patterns of abundance of these lipids in CL during luteal development, maintenance, and rescue, in any species. Therefore, the abundance of lipid mediators, including endocannabinoids and oxylipins from cyclooxygenase (COX), lipoxygenase (LOX), and cytochrome P450 (CYP)-dependent metabolism were profiled in the CL on days 4, 11, and 18 of the estrous cycle and on day 18 of pregnancy. The objectives of this study were to identify lipid mediators that regulate luteal function during these transitions, to integrate the lipid profile with a previously published mRNA profile of CL during maternal recognition of pregnancy, and to determine the effect of a subset of lipids on *in vitro*progesterone production.

## Methods

### Animals and Tissue Collection

All procedures were approved by the Penn State University Institutional Animal Care and Use Committee or The Ohio State University Animal Care and Use Committee. Regularly cyclic cows were observed for behavioral signs of estrus and the day of standing estrus was assigned as day 0. For the targeted metabolomics experiment, sixteen dairy cows were used, with four cows/group in four groups. For cows assigned to the day 4 group, upon observation of estrus and a dominant follicle by ultrasound, cows were given an injection of GnRH (Factrel, 100 μg; Zoetis) in order to precisely time ovulation relative to time of collection for these early CL. Cows were slaughtered on day 4 following estrus. For samples collected later than day 4, precise synchrony of ovulation relative to CL collection was not necessary, so no GnRH was given, and CL were collected via colpotomy. For CL of pregnancy, cows were bred by artificial insemination and a uterine flush was performed immediately following CL collection and was examined for embryo fragments to confirm the presence of a viable pregnancy. For all samples, tissue was snap frozen in liquid nitrogen immediately following tissue collection and stored at −80°C thereafter. For *in vitro* experiments, three to five dairy cows were used in each group, CL were collected on day 10–12 of the estrous cycle, and each treatment was applied to cells from each cow. Number of animals per group for these experiments is stated in the figure legend.

### Lipid Mediator Extraction and Analysis

Oxylipins and endocannabinoids were isolated using a Waters Ostro™ Sample Preparation Plate. Luteal samples were homogenized and 40 ± 8 mg were added to 2 mL polypropylene tubes spiked with a 5 μL antioxidant solution (0.2 mg/ml solution BHT/EDTA in 1:1 MeOH:water) and 10 μL 1,000 nM analytical deuterated surrogates as previously described (13, 14). Samples were then mixed with 35 μL methanol, 550 μL isopropanol w/10 mM ammonium formate, 1% formic acid and 100 μL water, and the tube was placed in a Geno/Grinder 2010 (SPEX SamplePrep) for 30 s and centrifuged at 10,000 × g for 5 min at room temperature. Supernatants were transferred into the Ostro plate wells and captured in glass inserts containing 10 μL of 20% glycerol in methanol by applying 15 mmHg of vacuum for 10 min. The eluent was dried under vacuum and reconstituted with 100 μL, 1:1 MeOH/ACN (v/v) containing 100 nM of 1-cyclohexyl ureido, 3-dodecanoic acid and 1-phenyl ureido, 3-hexanoic acid urea used as internal standards (gifts from Dr. B. D. Hammock, University of California, Davis). The samples were then vortexed and filtered at 0.1 μm through PVDF membranes (Millipore) by centrifugation <4,500 × g (rcf) for 3 min at 6°C. The filtrate was transferred to inserts in amber glass and stored at −20°C for <48 h before analysis by UPLC-MS/MS. Analytes in 5 μL extract aliquot were separated on a 2.1 × 150 mm, 1.7 μm Acquity BEH column (Waters) using published protocols for oxylipins and endocannabinoids (13, 15). All chromatographic and mass spectral acquisition parameters are provided in Supplemental Tables 1–4. Samples were held at 10°C. Separated residues were detected by negative mode electrospray ionization for oxylipins and positive mode electrospray ionization for endocannabinoids using multiple reaction monitoring on an API 6500 QTRAP (AB Sciex). Analytes were quantified using internal standard methods and 5- to 7-point calibration curves (*r*^2^ ≥ 0.997). Calibrants and internal standards were either synthesized [10,11-DHN, 10,11-DHHep, 10(11)-EpHep] or purchased from Cayman Chemical, Avanti Polar Lipids Inc., or Larodan Fine Lipids. Data was processed with AB Sciex MultiQuant version 3.0.2. The internal standards were used to quantify recovery of surrogate standards.

### Cell Culture and Progesterone Enzyme-Linked Immunosorbent Assays

For all cell culture experiments, CL from days 10–12 were used. CL were dissociated as previously described (16) and cells were put into culture in serum-free Hams F12 medium supplemented as described previously (16). For progesterone production experiments, all treatments were initiated on day 0. For assessment of progesterone on day 1, medium was collected 18–22 h after cells were initially put into culture. For assessment of progesterone on day 7, medium was changed and fresh treatments were added on days 1, 3, and 5, and medium was collected and assayed for progesterone on day 7. 5-KETE and 15-KETE (Cayman Chemical) were dissolved in EtOH, which was added to medium at a final concentration of 0.0001 and 0.001% v/v, respectively, for 0.1 and 1 ng/mL treatments. Concentrations of lipids were based on tissue concentration of 5-KETE and 15-KETE and calculated using the equation used in Pate (17), with the lower concentration, 0.1 ng/mL, being approximately physiological. Additional treatments were luteinizing hormone (LH; NHPP; 10 ng/mL), PGF2A (Sigma; 10 ng/mL) and LH + PGF2A. Progesterone enzyme-linked immunosorbent assays (ELISA) were performed as previously described for culture medium samples (18), with the following modifications: samples and antibodies were diluted in a buffer of 0.04 M 3-(N-morpholino)propanesulfonic acid, 0.12 M NaCl, 0.01 M EDTA, 0.05% Tween 20, 0.005% chlorhexidine digluconate, and 0.1% gelatin. Antibodies were Goat Antimouse IgG Antibody (2 ug/mL; EMD Millipore) and Monoclonal Progesterone Antibody (57.8 ng/mL; East Coast Bio). Progesterone conjugate (East Coast Bio) was diluted 1:750. Functional range of the progesterone standard curve (Cayman Chemical) was 0.16–10 ng/mL and all samples were diluted so that they fit within the functional range of the assay.

### Quantitative PCR

Luteal cells were allowed to adhere overnight, and on day 1, were treated with 0.1, 1, or 100 ng/mL of 5-KETE. 5-KETE was dissolved in ethanol, making final concentrations of ethanol 0.0001, 0.001, and 0.1% v/v, respectively. Cells were collected after 24 h of treatment. In another experiment, luteal cells were treated with 100 ng/mL of 5-KETE for 7 days prior to collection. Quantitative PCR (qPCR) was performed as described previously for cultured luteal cells (19), with the exception that RNA was isolated using the Qiagen RNeasy Plus Mini kit following instructions from the manufacturer.

### Statistical and Pathway Analysis

All statistical analysis of targeted metabolomics data was performed using JMP Pro v 13.0 (SAS Institute). Although each experiment was analyzed separately, the same method of analysis was used for each. For each metabolite, normality was assessed using the Shapiro-Wilk test and a Box Cox transformation to obtain normal distributions when distributions that were not normal were encountered. Normal data were then assessed for outliers per treatment group (*N* = 4) using a 95% Dixon test. If detected, outliers were removed from the raw data, missing data was imputed per treatment group using the multivariate singular value decomposition method (20), and the Box Cox transformation was repeated. Students *t*-test or ANOVA with a Tukey *post-hoc* test were used to assess the effect of stage of cycle or pregnancy on lipid abundance for each lipid measured. In all cases, statistical significance was considered when *P* < 0.05. For the experiment comparing early pregnancy to the late estrous cycle, statistical tendencies (*P* < 0.10) were few and significant differences were even fewer. Because the small number of observations per group (*N* = 4/group) resulted in low power, values approaching significance were considered in pathway analyses, though this cutoff was only used for the latter experiment. False discovery rate-adjusted *P*-values (Padj) were also calculated (21) with a false discovery rate of 20%, and are available in Tables 1, 2. However, these adjusted *P*-values were not used to select lipids used in pathway analysis, both because of the low power in this study and because this was a targeted metabolomics study, with relatively few molecules measured, as compared to an untargeted transcriptomics or proteomics study. Principal component analysis was used to determine whether differences among groups within an experiment allowed groups to be distinguished individually. Finally, to identify groups of lipids with similar patterns of expression, hierarchical cluster analysis was performed on lipids from the estrous cycle experiment only. Pathway analysis was conducted in Ingenuity Pathway Analysis (IPA; Qiagen). General lipid pathway information, including precursor, metabolism, and nomenclature information, was obtained from www.hmdb.ca and pubchem.ncbi.nlm.nih.gov. These data are available online as Study ST001245 at the Metabolomics Workbench (https://www.metabolomicsworkbench.org/).

**Table 1:** Differentially abundant lipids from CL of days 4, 11, and 18 of the estrous cycle.

**Differ between**	**Cluster**	**Common name**	**Chemical Class**	**General pathway classification**	**Log2 fold change day 11 vs. 4**	**Log2 fold change, day 18 vs. 4**	***P*-value**	***P*-adj^a^**
Days 4 and 11	5	5-KETE^b^	Ketone	Lipoxygenase/alcohol dehydrogenase	1.43	1.31	<0.001	0.001
		2-Arachidonylglycerol	Acylglycerol	Endocannabinoid	1.49	1.19	0.011	0.085
	6	19,20-DiHDPA	Vicinal diol	Cytochrome P450/epoxide hydrolase	2.98	4.14	0.006	0.085
		17,18-DiHETE	Vicinal diol	Cytochrome P450/epoxide hydrolase	2.56	3.39	0.009	0.085
		5,15-DiHETE	Non-vicinal diol	Lipoxygenase	3.42	6.30	0.021	0.105
	7	8(9)-EpETrE	Epoxide	Cytochrome P450	3.61	4.86	0.006	0.085
		Linoleic acid	PUFA	Fatty acid	0.93	1.41	0.007	0.085
		Adrenoylethanolamide	Acylethanolamide	Endocannabinoid	1.76	4.20	0.008	0.085
		9,12,13-TriHOME	Triol	Autooxidation	1.38	2.91	0.009	0.085
Days 4 and 18	7	11(12)-EpETrE	Epoxide	Cytochrome P450	2.94	4.29	0.010	0.085
		12(13)Ep-9-KODE^b^	Epoxy ketone	Lipoxygenase	1.56	3.06	0.010	0.085
		Dihomo-γ-Linolenoyl ethanolamide	Acylethanolamide	Endocannabinoid	1.06	3.97	0.012	0.085
		15,16-DiHODE	Vicinal diol	Cytochrome P450/epoxide hydrolase	2.15	3.86	0.016	0.105
		2-Linoleoylglycerol	Acylethanolamide	Endocannabinoid	1.52	3.35	0.018	0.105
		9(10)EpO (epoxystearate)	Epoxide	Cytochrome P450	1.18	2.53	0.023	0.105
		Stearoylethanolamide	Acylethanolamide	Endocannabinoid	1.72	2.06	0.028	0.105
		14(15)-EpETrE	Epoxide	Cytochrome P450	2.50	3.85	0.030	0.105
		15-KETE^b^	Ketone	Lipoxygenase/alcohol dehydrogenase	1.08	1.30	0.030	0.105
		Arachidonoylethanolamide	Acylethanolamide	Endocannabinoid	0.82	2.76	0.031	0.105
		9(10)-EpOME	Epoxide	Cytochrome P450	1.96	3.72	0.039	0.105
		6-trans-Leukotriene B4	Non-vicinal diol	Lipoxygenase	1.47	3.78	0.044	0.105
		8-HETE	Alcohol	Lipoxygenase	1.25	3.95	0.044	0.105
		Arachidonic acid	PUFA	Fatty acid	0.85	1.37	0.044	0.105

a False Discovery Rate adjusted p-values, for false discovery rate of 20%.

b *Indicates a lipid that was also differentially abundant during maternal recognition of pregnancy*.

**Table 2:** Differentially abundant lipids, and those that tended to be differentially abundant, in CL of day 18 of the estrous cycle and pregnancy.

**Common name**	**Chemical class**	**General pathway classification**	**Log2 fold change (pregnant vs. cyclic)**	***P*-value**	***P*-adj^a^**
15-KETE^b^	Ketone	Lipoxygenase/alcohol dehydrogenase	−1.57	<0.001	0.008
5-KETE^b^	Ketone	Lipoxygenase/alcohol dehydrogenase	−0.69	0.063	0.888
12(13)Ep-9-KODE^b^	Epoxy ketone	Lipoxygenase	−1.99	0.083	0.888
alpha-linolenoyl ethanolamide	Acylethanolamide	Endocannabinoid	−3.64	0.084	0.888
15(16)EpODE	Epoxy ketone	Cytochrome P450	−1.70	0.090	0.888

a *False Discovery Rate adjusted p-values, for false discovery rate of 20%*.

b *Indicates a lipid that was also differentially abundant during the estrous cycle*.

The dataset of lipids that changed during maternal recognition of pregnancy was integrated with a transcriptomics dataset in which CL of the cycle and pregnancy were compared on day 17. In this study, *N* = 4/group and 140 differentially abundant mRNA were identified (19). These data are available online the NCBI's Gene Expression Omnibus [(22); https://www.ncbi.nlm.nih.gov/geo/], accession number GSE135342.

All statistical analysis for progesterone production and qPCR experiments were performed in SAS 9.4 (SAS Institute). Animal was included as an effect in the model, significance was considered at *P* < 0.05, and for the qPCR experiment, the reference gene, RPL19, was used as a covariate to account for differences in RNA loading and cDNA synthesis.

## Results

### Lipid Mediator Profile

**Figure 1:**
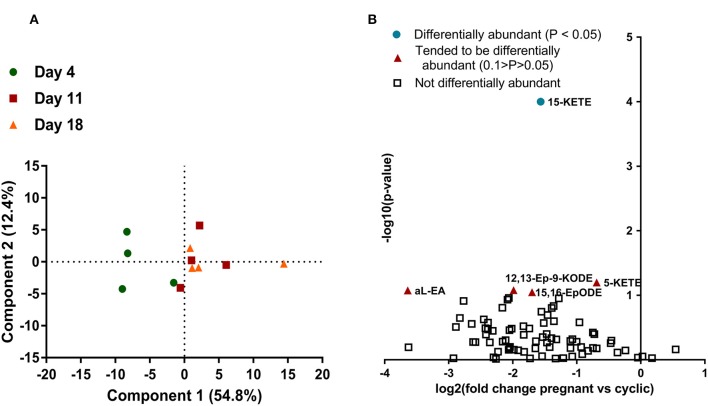
Lipids in the CL during the estrous cycle and early pregnancy (*N* = 4/group). **(A)** Principle component analysis, with each point representing one sample from the lipid profiling experiment. Samples from day 4 are represented with a green circle, samples from day 11 are represented with a red square, and samples from day 18 are represented by an orange triangle. Distance apart on principle components 1 and 2 is a proxy for overall similarity in lipid abundance profile, with closer points being more alike. **(B)** Volcano plot of all lipids measured in the CL on day 18 of the estrous cycle and pregnancy. Each lipid is represented by a point, with the lipids that did not change represented with an open square, the lipids that tended to change represented by an orange triangle, and the lipids that changed (*P* < 0.05) represented by a teal circle. Magnitude of statistical significance is represented in the y-axis, while numerical fold change is represented on the x-axis, with lipids that were greater during the CL of the estrous cycle on the left and lipids that were greater in the CL of pregnancy on the right.

Seventy-nine lipids were measured in the CL, at four times, day 4, 11, 18 of the estrous cycle, and day 18 of pregnancy (Supplemental Table 5). For lipids measured during the estrous cycle, principle component analysis (PCA) was used to reduce the 79 variables into linearly uncorrelated principle components. Only component 1 meaningfully separated the data, explaining 54.4% of the variation in the dataset. The day 4 samples clustered separately from day 11 and 18 samples along component 1, though 1 day 4 sample was more similar to the day 11 and 18 samples than the other 3 day 4 samples. The day 11 and 18 samples did not cluster separately. Analysis of component 1 scores indicated that the day 4 samples differed from the day 11 and 18 groups, but those groups did not differ from each other (Figure 1A).

Samples from day 18 of the estrous cycle and pregnancy did not cluster separately when PCA was performed (data not shown). The majority of lipids were less abundant on day 18 of pregnancy than day 18 of the estrous cycle, although this numerical change was small and only resulted in a few lipids that were significantly differentially abundant (Figure 1B).

### Estrous Cycle-Associated Changes

**Figure 2:**
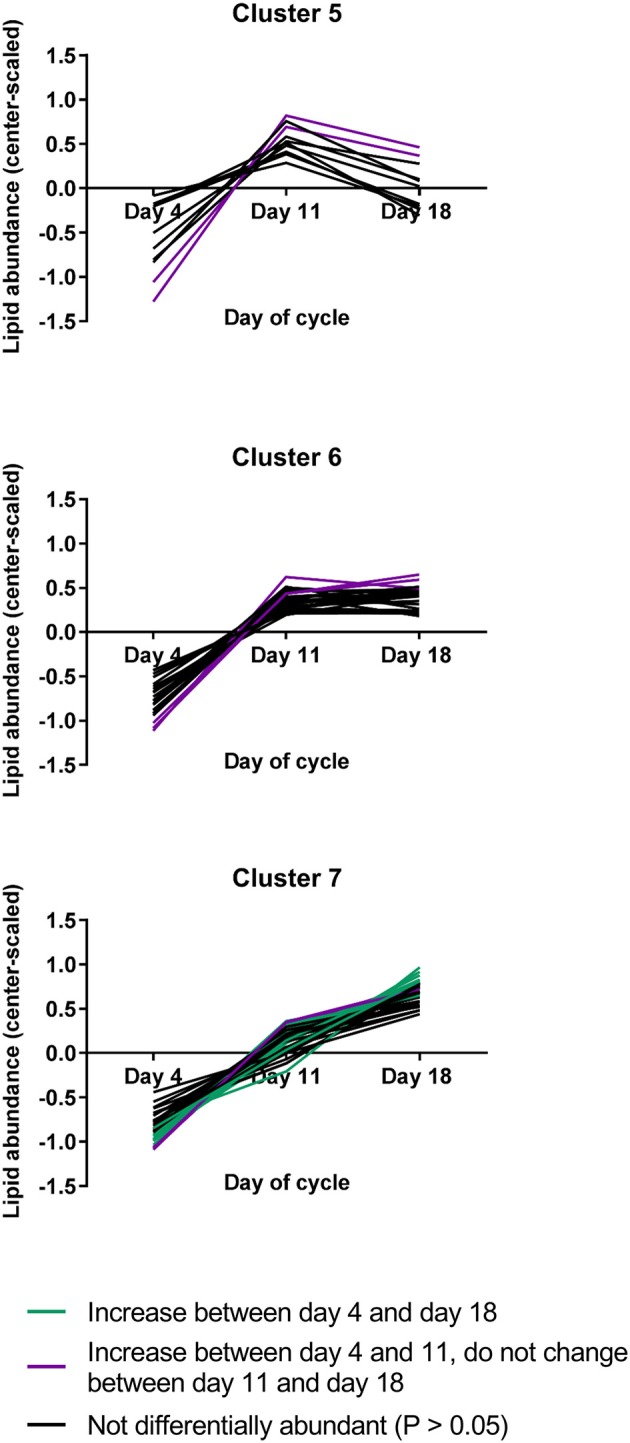
The three lipid abundance clusters that included lipids that changed during the estrous cycle (*N* = 4/group). Lipids that appeared to change but were not statistically differentially abundant are represented by black lines, while lipids that changed between days 4 and 11 are represented by purple lines and lipids that changed between days 4 and 18 are represented by turquoise lines.

Cluster analysis was used to determine which lipids had similar patterns of abundance during the estrous cycle, regardless of the detection of a measurable change (Supplemental Table 5). Seven clusters were generated, among which three clusters contained 73 of the 79 lipids measured, including all differentially abundant lipids (Figure 2). The remaining clusters contained 1 or 2 lipids each, none of which changed (Supplemental Figure 1). The cluster with the greatest number of lipids was cluster 7, a cluster in which lipids appeared to increase from day 4 through day 18.

Twenty-three lipids were differentially abundant as the estrous cycle progressed and all increased over time. Among these, fourteen were greater on day 18 than on day 4, with those on day 11 not differing from either of the other two groups. These lipids were all in cluster 7. For nine metabolites, abundances were greater on day 11 than on day 4 and remained greater on day 18. These lipids were in clusters 5, 6, and 7. Among the lipids that were differentially abundant during the estrous cycle, 20 were uniquely differentially abundant only in the estrous cycle, while three were also differentially abundant or tended to be differentially abundant during maternal recognition of pregnancy: 5-KETE, 15-KETE, and 12(13)Ep-9-KODE (Table 1). The lipids that changed during luteal development and maintenance may be considered in groups of lipids derived from common precursors or in groups synthesized by common enzymes or enzyme groups.

Concentrations of luteal fatty acid epoxides, bioactive products of cytochrome (CYP) P450 metabolism, were altered over the estrous cycle, as were their enzymatic hydrolysis products, the 1,2- or vicinal carbon diols (Table 1). Between days 4 and 11 increases were observed in the arachidonate-derived 8(9)-epoxyeicosatrienoic acid (EpETrE), diols from the docosahexenoate-derived diol, 19,20-dihydroxydocosapentenoic acid (DiHDPA), the putative oxidative stress marker 9,12,13-trihydroxyoctadecenoicacid (TriHOME) from linoleate, along with linoleate itself. Between days 4 and 18, 11(12)- and 14(15)-EpETrE concentrations increased, along with the linoleate-derived 9(10)-epoxyoctadecenoic acid (9(10)-EpOME), and the oleate-derived 9(10)-epoxyoctadecanoic acid (EpO). Alpha-linolenate-derived 15,16-dihydroxyoctadecadienoic acid (15,16-DiHODE) also increased at this time.

The two classical endocannabinoids, arachidonoylethanolamide (anandamide) and 2-arachidonylglycerol changed, with anandamide increasing between days 4 and 18, while 2-arachidonylglycerol increased between days 4 and 11. Several related N-acylethanolamides also changed during the luteal phase, including adrenoylethanolamide, dihomo- γ-linolenoyl ethanolamide, and stearoylethanolamide (Table 1).

**Figure 3:**
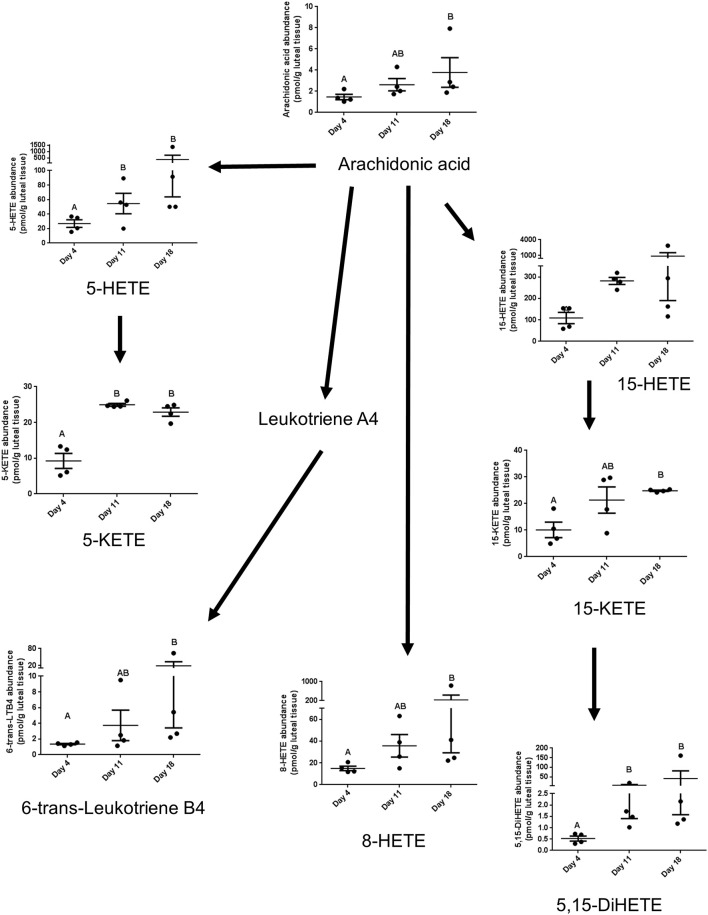
The lipids that are components of the lipoxygenase pathway that were measured in this study, with arrows indicating synthesis relationships between lipids. 6-trans-LTB4 is also a reported decomposition product of the 5-lipoxygenase derived leukotriene C4.

The lipoxygenase pathway results in production of lipoxins and leukotrienes. 8-HETE increased between day 4 and 18, 5-HETE tended to increase between days 4 and 11, 18 (*P* = 0.06), and 15-HETE did not change. On the other hand, the alcohol dehydrogenase dependent ketone metabolites of these alcohols were strongly affected by estrous cycle stage. 5-KETE, derived from 5-HETE, changed between days 4 and 11, while 15-KETE, derived from 15-HETE, changed between days 4 and 18. The linoleate-derived 12(13)-epoxy-9-ketooctadecadienoicacide (12-(13)Ep-9-KODE) changed between days 4 and 18. Concentrations of 6-trans-leuktriene B4 (6-trans-LTB4), a reported decomposition product of the 5-lipoxygenase derived leukotriene C4, also increased between days 4 and 18 (Table 1, Figure 3).

**Figure 4:**
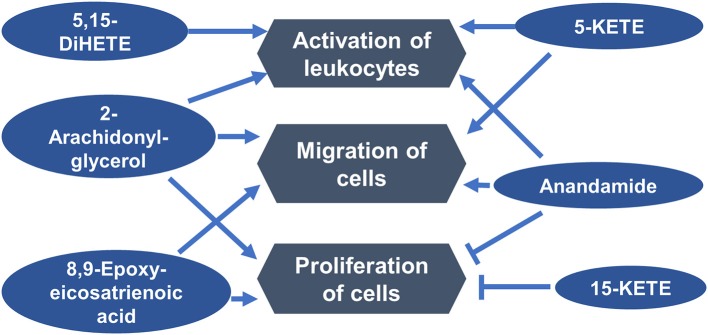
Luteal metabolomics and pathway analysis during the estrous cycle. A functional network showing top functions in which differentially abundant lipids from the estrous cycle may be involved.

Ingenuity Pathway Analysis (IPA; Qiagen) was used to determine functions in which differentially abundant lipids from the estrous cycle may be involved. One functional network, including the functions that were associated with the greatest number of lipids, indicated that six differentially abundant lipids may be regulators of immune cell activation, migration, and function (Figure 4).

### Pregnancy-Associated Changes

**Figure 5:**
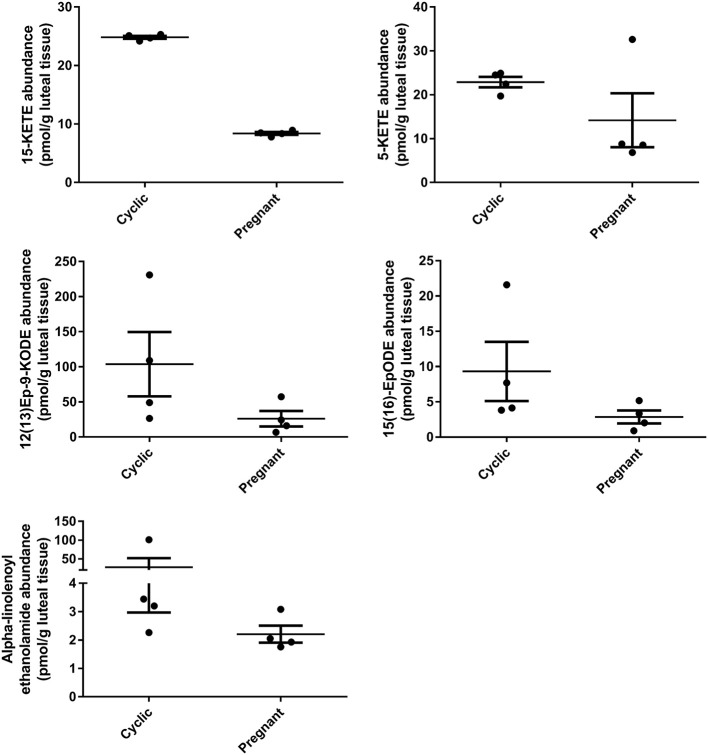
Luteal metabolomics during maternal recognition of pregnancy (*N* = 4/group). Lipids that changed (15-KETE; *P* < 0.05) or tended to change (*P* < 0.1) during maternal recognition of pregnancy.

During early pregnancy, one lipid mediator, 15-KETE, declined, while four others tended to decline, including 5-KETE, 12(13)Ep-9-KODE, 15(16)EpODE, and alpha-linolenoyl ethanolamide (Figure 5). 5-KETE, 15-KETE, 12(13)-Ep-9-KODE are all lipoxygenase-derived metabolites as described above. The alpha-linolenic acid metabolites 15(16)-EpODE from CYP metabolism, and alpha-linolenoyl ethanolamide, an endocannabinoid-like compound were also changed (Table 2, Figure 5).

**Figure 6:**
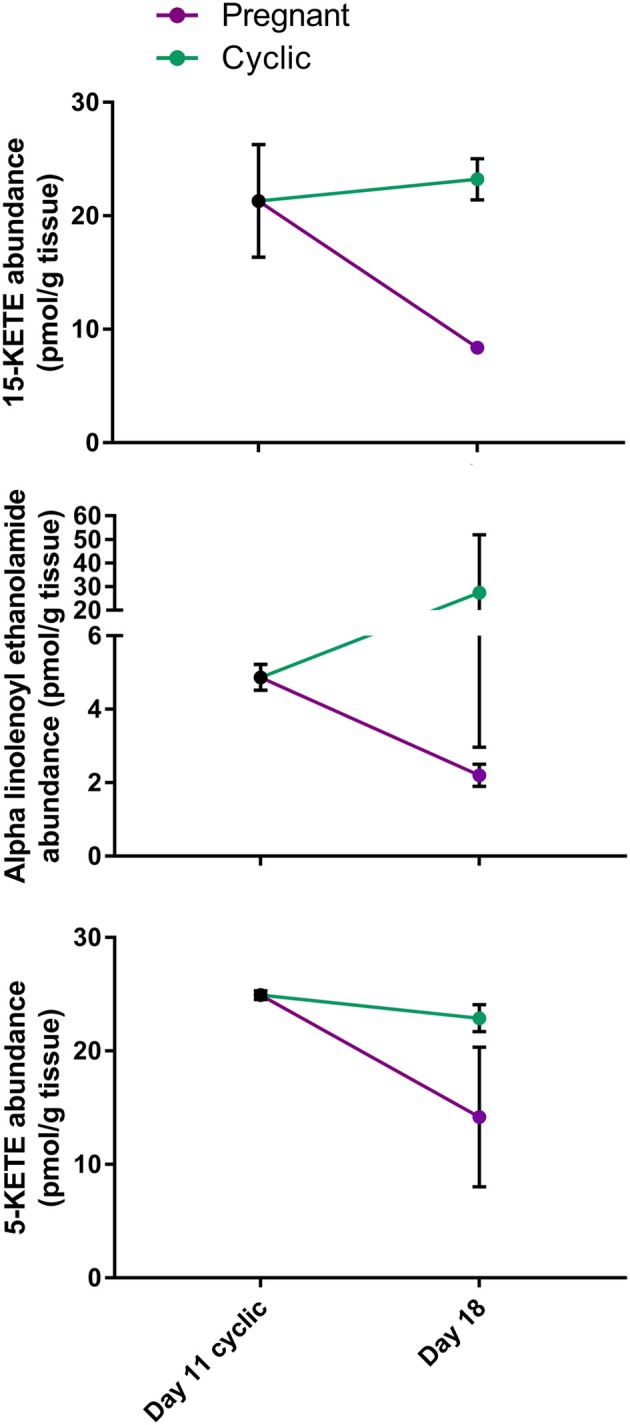
Comparison of lipid abundance on day 11 of the estrous cycle and day 18 of pregnancy. Mean lipid abundance on day 11 is represented by a black circle, with day 18 of the estrous cycle represented in teal and day 18 of pregnancy represented in purple. 15-KETE and alpha-linolenoyl ethanolamide differed (*P* < 0.05) when day 11 of the estrous cycle and day 18 of pregnancy were compared, while 5-KETE demonstrated a similar numerical pattern (*P* = 0.11).

In the comparison of CL from day 18 of the cycle and pregnancy, it is difficult to determine whether changes observed result from exposure of the CL of the cycle to early pulses of PGF2A from the uterus or to conceptus signaling. Therefore, to determine in which group the lipid changed, day 11 CL were compared to CL of pregnancy, only for lipids that changed or tended to change during early pregnancy. Remarkably, 15-KETE and alpha-linolenoyl ethanolamide, which did not change between days 11 and 18, were both less in the CL of pregnancy than on day 11 (*P* < 0.05), indicating that they may be downregulated during early pregnancy. A very similar pattern was observed for 5-KETE, though 5-KETE did not differ from day 11 significantly (*P* = 0.11; Figure 6). For the other two lipids that tended to change during early pregnancy, no difference between day 11 and pregnancy could be detected.

**Figure 7:**
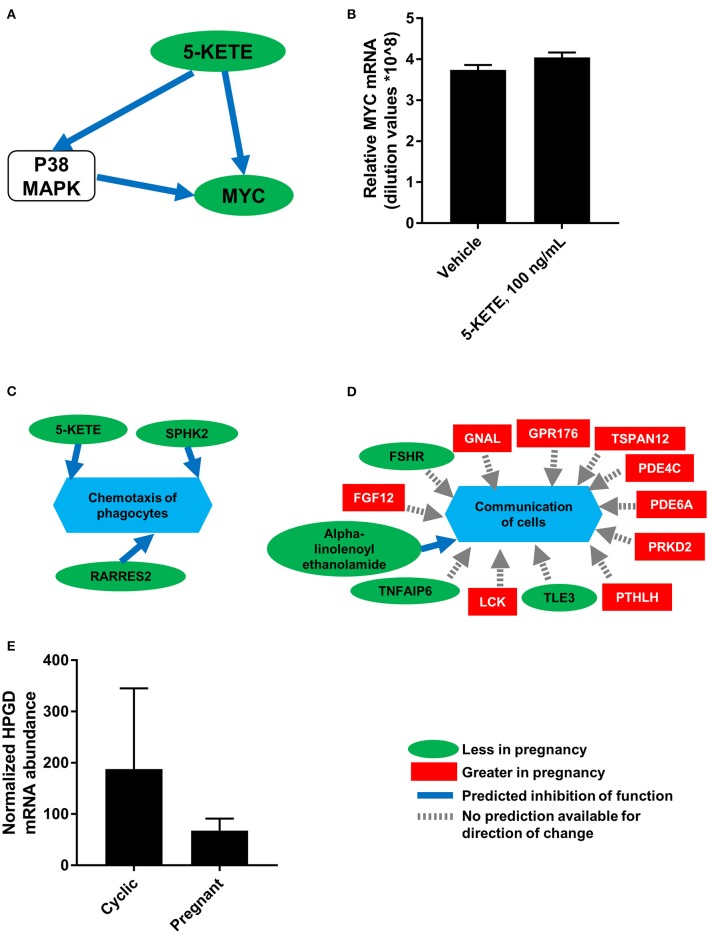
Integration of luteal transcriptomics and metabolomics during maternal recognition of pregnancy [*N* = 4/group for metabolomics and transcriptomics; (19)]. **(A)** The network from IPA indicating a relationship between 5-KETE and MYC, with a green oval indicating less abundance in the CL of pregnancy, a blue arrow indicating predicted inhibition, and a white rounded square indicating a molecule that was not measured. **(B)** Effect of 5-KETE on MYC mRNA abundance in cultured luteal cells (*N* = 3). **(C,D)** Predicted functions of differentially abundant lipids and mRNA from maternal recognition of pregnancy, with a green oval indicating less abundance in the CL of pregnancy, a red square indicating greater abundance in the CL of pregnancy, a blue arrow indicating predicted inhibition, and a gray dashed arrow indicating that no prediction is available for direction of change. **(E)** HPGD abundance on day 17 of the estrous cycle and pregnancy, as measured by transcriptomics; *P* = 0.11.

To generate a more comprehensive profile of luteal changes during maternal recognition of pregnancy, mRNA that were differentially abundant during maternal recognition of pregnancy (19) were integrated with the lipids from this study. Analysis in MetaboAnalyst (23) and in Reactome (24) demonstrated no significantly modulated pathways that included both differentially abundant mRNA and differentially abundant lipids. This indicated no clear enzyme-lipid or lipid-receptor interaction as a result of integration. However, network analysis in IPA (Qiagen) indicated that networks “Lipid metabolism, molecular transport, and small molecule biochemistry” and “DNA replication, recombination and repair, cell death and survival, cellular function and maintenance” involved both differentially abundant lipids and mRNA. Further, these networks indicated that 5-KETE, which tended to be differentially abundant during maternal recognition of pregnancy, may be associated with ERK1/2 and P38 MAPK signaling. One network indicated that 5-KETE may regulate expression of MYC proto-oncogene (*MYC;* Figure 7A), which was greater in the CL of the estrous cycle relative to the CL of pregnancy. Therefore, cultured luteal cells were treated with 100 ng/mL 5-KETE for 24 h, to determine if 5-KETE regulates *MYC* in luteal cells (Figure 7B). Concentrations of 1 and 0.1 ng/mL for 24 h and 100 ng/mL for 7 days were also tested (data not shown). 5-KETE did not alter *MYC* in cultured luteal cells at any concentration. Functional analysis in IPA indicated that a decrease in 5-KETE, as well as sphingosine kinase 2 and retinoic acid receptor responder 2, may regulate a decrease in chemotaxis of phagocytes during early pregnancy (Figure 7C). Additionally, a number of differentially abundant mRNA and alpha-linolenoyl ethanolamide may together be regulators of cellular communication in the CL (Figure 7D).

Notably, the precursor of 15-KETE, 15-HETE, was not different during maternal recognition of pregnancy. In the transcriptomics dataset comparing CL of day 17 of the estrous cycle and pregnancy, the mRNA encoding the enzyme responsible for synthesis of 15-KETE, hydroxyprostaglandin dehydrogenase 15-(NAD; *HPGD*), was numerically less (*P* = 0.11) in the CL of pregnancy (Figure 7E).

### Luteal Progesterone Production in Response to KETEs

**Figure 8:**
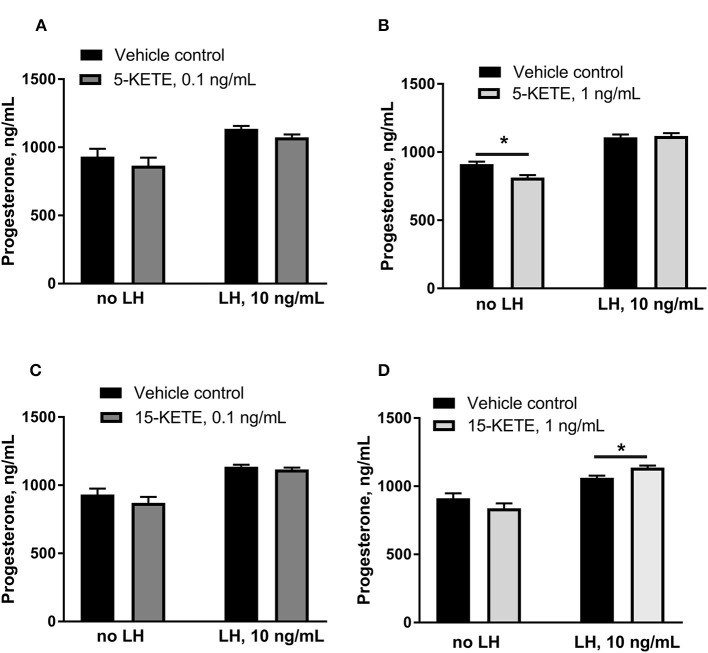
Effect of 5-KETE and 15-KETE on progesterone production in cultured luteal cells on day 1 of culture. Significant differences (*P* < 0.05) between lipid-treated and vehicle control are denoted with a *. The effect of 0.1 ng/mL **(A)** or 1 ng/mL **(B)** 5-KETE and of 0.1 ng/mL **(C)** or 1 ng/mL **(D)** 15-KETE (*N* = 5 and *N* = 4 for without and with LH groups, respectively).

5-KETE and 15-KETE changed during the estrous cycle and during maternal recognition of pregnancy and were identified as likely key regulators of physiological functions in luteal cells. To assess effects of these two lipids, luteal cells were cultured with 5-KETE or 15-KETE. On day 1 of culture, 0.1 ng/mL 5-KETE did not alter progesterone in the absence or presence of LH (Figure 8A), while 1 ng/mL 5-KETE reduced luteal progesterone, but only in the absence of LH (Figure 8B). Conversely, while 0.1 ng/mL 15-KETE had no effect (Figure 8C), 1 ng/mL 15-KETE induced progesterone only in the presence of LH (Figure 8D).

**Figure 9:**
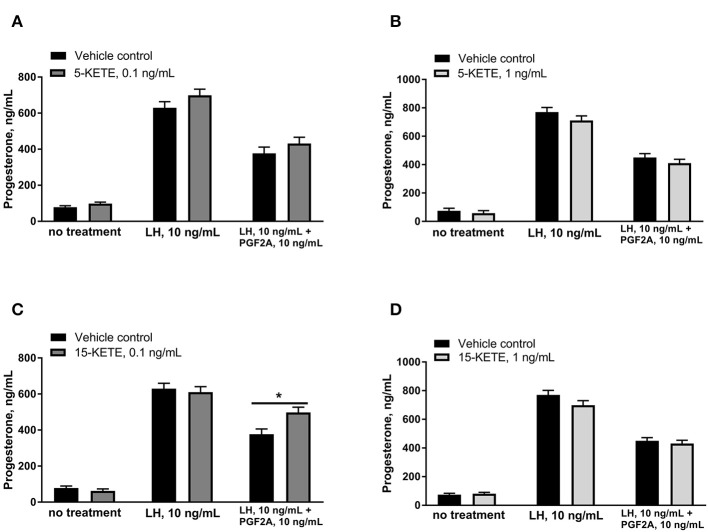
Effect of 0.1 ng/mL **(A)** and 1 ng/mL **(B)** 5-KETE and of 0.1 ng/mL **(C)** and 1 ng/mL **(D)** 15-KETE on progesterone production in cultured luteal cells on day 7 of culture. Significant differences (*P* < 0.05) between lipid-treated and vehicle control are denoted with a * (*N* = 4).

Effects of 5-KETE and 15-KETE were also assessed on day 7 of culture, in order to determine whether these lipids altered the PGF2A-induced inhibition of LH-response seen in luteal cells on day 7 of culture (25). 5-KETE did not have any effect on day 7, either alone or in combination with LH or LH + PGF2A (Figures 9A,B). However, 0.1 ng/mL 15-KETE reduced the degree to which PGF2A inhibited LH-stimulated progesterone production (Figure 9C), whereas 1 ng/mL 15-KETE did not have this effect (Figure 9D).

## Discussion

The objective of this study was to identify novel lipids that may be either supportive of or deleterious to luteal function and progesterone production. *In silico* pathway analysis indicated that differentially abundant lipids from the metabolic profiling experiment may regulate functions associated with luteal cell-immune cell communication and cell survival and proliferation. Moreover, this profiling allowed identification of lipid regulators of luteal progesterone production and response to PGF2A *in vitro*.

The endocannabinoid family of molecules activate the cannabinoid receptors, CNR1 and CNR2, both G protein-coupled receptors (26). These molecules are derived from membrane phospholipids and include both N-acylethanolamines (including the classical endocannabinoid, anandamide) and 2-acylglycerols (including the classical endocannabinoid, 2-arachidonylglycerol). Members of each of these classes increased over time during the cycle and one more tended to be less in the CL of pregnancy.

CL express the cannabinoid receptors CNR1 and CNR2, as well as fatty acid amide hydrolase (FAAH), which is responsible for the breakdown of endocannabinoids (27). *In vitro* activation of CNR1 or 2, or inhibition of FAAH, reduced progesterone, PGF2A, and PGE1 (28). Importantly, activation of CNR1 and 2 *in vivo* reduced plasma progesterone, luteal weight, and LH receptor abundance (29). Moreover, in CNR1 knockout mice, the luteolytic effect of LPS was lost, with no increase in luteal cyclooxygenase enzymes or PGF2A content, indicating that the mechanism by which LPS induced luteal regression requires endocannabinoid signaling (30). Although only one endocannabinoid, alpha-linolenoyl ethanolamide, tended to be less abundant in the CL of pregnancy, this change may contribute to mechanisms that ensure luteal survival.

Progesterone increased abundance of FAAH in T lymphocytes (31), indicating that perhaps the high progesterone content of the CL may promote metabolism of endocannabinoids, while the luteolytic cytokine, IFNG (32, 33), decreased abundance of FAAH (31). In addition, stearoylethanolamide has been documented as a proapoptoic factor, particularly in combination with nitric oxide (34). Perhaps the increase in stearoylethanolamide at the end of the estrous cycle makes luteal cells more susceptible to apoptosis during luteal regression. Together, these observations lead to the speculation that increased endocannabinoid abundance could promote luteal regression.

Linoleic acid is an essential fatty acid, meaning that it must be derived from the diet. While little is known about luteal effects of the linoleic acid metabolites measured in this study, linoleic acid itself has been studied extensively and has been demonstrated to decrease PGF2A and PGE2 production by cultured luteal cells, without altering progesterone (35). Feeding a source of omega-6 fatty acids increased linoleic acid content of CL and improved pregnancy rates in beef cattle (36). Given that linoleic acid must be derived from the diet, perhaps luteal tissue uptake and storage of linoleic acid is enhanced between days 4 and 11, to support luteal function or, since free fatty acids were measured, perhaps there is an increase in phospholipase activity and an increase in liberation of linoleic acid from cell membranes.

The three physiologically stable EpETrEs increased in CL as the estrous cycle progressed. These lipids are short-lived vascular inhibitors of functions such as vasoconstriction (37, 38) and blood clotting (39). Their increase over time may enhance blood supply to the CL, thus supporting luteal function. The high rate of progesterone production in the CL requires a large supply of oxygen, and every steroidogenic cell is in contact with a blood vessel.

Surprisingly, no differences were observed in tissue concentrations of PGF2A, PGE2, or 6-keto PGF1A, the stable metabolite of prostacyclin. In a previous report, PGF2A and 6-keto PGF1A content of freshly dispersed luteal cells was measured and reported to be greater on day 5 than on days 10, 15, or 18, with a numerical decline in ratio of 6-keto PGF1A to PGF2A over time (40). A similar decline in ratio was observed here, with ratios of 5.5, 2.3, and 3.2 on days 4, 11, and 18. This supports the results of Milvae and Hansel (40) and indicates that had the number of animals per group in this study been increased and animal-to-animal variation had been reduced, this study would have similar findings. Although Lee et al. (7) reported less intraluteal PGF2A and greater PGE2 on day 16 of ovine pregnancy relative to the same day of the estrous cycle, these CL were likely already regressing on day 16, making comparison with the findings presented here irrelevant. Lukazewska and Hansel (1) reported greater free arachidonic acid concentration in CL of the estrous cycle than CL of pregnancy. In this study, arachidonic acid was slightly greater, although the number of animals per group was more than four times less than in the study of Lukazewska and Hansel (1).

Several lipids of the lipoxygenase pathway changed, both during the estrous cycle and during maternal recognition of pregnancy. LTB4 has previously been reported to increase both PGE2 and progesterone production by luteal cells (9). Lukaszewska and Hansel (1) reported a relatively linear increase in serum progesterone until the time of luteolysis, which may be supported in part by the luteotrophic effects of LTB4, as it increases during this time.

Members of the lipoxygenase pathway, including 5- and 15-KETE, may regulate infiltration of immune cells. C-C motif chemokine ligand 2 (CCL2), which is an important regulator of immune cell infiltration into the CL (41, 42) and luteal response to PGF2A (43), acted synergistically with 5-KETE to attract monocytes (44). Moreover, the pattern of change in 5-KETE abundance in this experiment is quite similar to the pattern of change in CCL2 in the CL on similar days (45). 15-KETE has been reported to have antiinflammatory properties (46), and indirectly induced immune cell infiltration by increasing abundance of E-selectin on endothelial cells and thus monocyte adhesion (47).

Luteal steroidogenic cells are potent activators of T cells (18), the luteal microenvironment programs resident T cells (48, 49), and alteration in activation state of luteal T cells has been implicated in both luteal regression (50) and luteal rescue (19). Pathway analysis using IPA indicated that four differentially abundant lipids, including 5-KETE and 5,15-DiHETE, and two endocannabinoids, anandamide and 2-arachidonyl-glycerol may regulate activation of leukocytes. Perhaps one mechanism of the ability of luteal steroidogenic cells to alter the function of T cells is through production of paracrine lipid mediators.

It has been reported that 5-KETE elicits its effect on cells by binding to oxoeicosanoid receptor 1 (*OXER1*), a G-protein coupled receptor (51). 5-KETE inhibits cAMP (52), induces calcium influx (53), and induces phosphorylation of p38 MAPK (54), all effects that could lead to the slight but significant 5-KETE-regulated reduction of progesterone observed in the current study. In the presence of LH, however, 5-KETE could not alter progesterone production. Because LH is a potent activator of cAMP, perhaps the moderate effect of 5-KETE was lost in the presence of this potent cAMP activator. 5-HETE, the molecule directly upstream from 5-KETE, inhibited luteal progesterone production in the presence and absence of LH (8, 55). However, in another report, 5-KETE induced StAR and steroid hormone production in adrenal and Leydig cell lines (56), though the mechanism of activation is not clear. The discrepancy between the study of Cooke et al. and this study may be explained by their use of cell lines, rather than the primary cells used here, or by the very high concentration of 5-KETE used in their study.

15-KETE increased progesterone production marginally but significantly, only in the presence of LH. Little is known about the pharmacology of 15-KETE and its receptor has not been identified. However, when cells were treated with a lipoxygenase inhibitor, which inhibited progesterone production, 15-HETE, the direct precursor of 15-KETE, induced progesterone only in the presence of LH, while 5-HETE and 12-HETE had no effect (57). 15-KETE, which increased between day 4 and 18, may also contribute to the change in LH responsiveness observed in luteal cells from older CL relative to younger CL. While LH induced an approximate doubling of progesterone production in dispersed luteal cells from day 5 and 10 of the estrous cycle, it induced a tripling of progesterone over basal in luteal cells from days 15 and 18 of the cycle (40).

During early pregnancy, luteal resistance to PGF2A transiently increases (4, 58, 59). Though this resistance may be due to exposure of CL to IFNT (60, 61), the mechanism of luteal resistance to PGF2A during early pregnancy has eluded researchers. Here, luteal cells treated with the lesser, but not the greater, concentration of 15-KETE, demonstrated reduced ability of PGF2A to inhibit LH response. This indicated a concentration-dependent response of luteal cells to 15-KETE, which was less in the CL of pregnancy. Perhaps the decline in 15-KETE during early pregnancy mediates an increased luteal resistance to PGF2A at that time, consistent with reports of other lipids altering luteal PGF2A response (12).

An *in silico* prediction, based on known regulatory relationships (62) indicated that during early pregnancy, 5-KETE may be a regulator of the transcription factor *MYC* and thus regulate the balance between cell survival and death (63). Despite testing multiple concentrations and times of treatment, no change was detected in *MYC* abundance as a result of 5-KETE treatment. This indicates that the change in luteal concentration of 5-KETE is not driving the change observed in *MYC* during early pregnancy, highlighting the importance of *in vitro* confirmation of *in silico* predictions.

Pathway analysis in IPA predicted that lipids may be regulators of cell proliferation in the CL. Early in the luteal phase, cell proliferation is so rapid that the CL had been compared to the fastest growing tumor. However, proliferation is turned off completely and the size and vascularity of the CL remains constant later in the cycle (64). Notably 15-KETE (65, 66) and anandamide (67) were predicted inhibitors of cell proliferation, including vascular endothelial cell proliferation, in the analysis in IPA. In addition, 5-KETE had a concentration-dependent effect on proliferation in cancer cell lines; high concentrations of 5-KETE inhibited proliferation via the peroxisome proliferator activated receptor gamma pathway (65). Perhaps the lesser concentrations of 5-KETE and 15-KETE in early pregnancy and their increase over time during the estrous cycle mediate the increased bloodflow to the CL during early pregnancy (68) and the decrease in cell proliferation in midcycle CL (64).

In conclusion, targeted metabolomic analysis of luteal tissue lipid mediators was used to identify 5-KETE and 15-KETE as potential key regulators of luteal function during important transitions in the luteal lifespan. Pathway analysis programs predicted lipid involvement in cell migration and chemotaxis, immune cell activation, and regulation of cell proliferation. *In vitro* experiments demonstrated that 5-KETE and 15-KETE were regulators of luteal progesterone and response to PGF2A, but not *MYC* abundance. Lipid regulation of luteal functions is an area that merits further investigation, as these lipids could be key paracrine regulators of luteal steroidogenic, endothelial, and immune cell functions.

## Data Availability Statement

All datasets generated for this study are included in the manuscript/Supplementary Files.

## Ethics Statement

The animal studies were reviewed and approved by the Pennsylvania State University IACUC or the Ohio State University IACUC.

## Author Contributions

CH performed integrated omics and pathway and statistical analyses, all *in vitro* experiments, designed the *in vitro* experiments, and wrote the manuscript. JP designed the targeted metabolomics experiments and was involved in experimental design of the *in vitro* experiments, and manuscript preparation. JN and RB performed the LC-MS/MS analyses, performed statistical analyses of targeted metabolomics data, and were involved in manuscript preparation.

### Conflict of Interest

The authors declare that the research was conducted in the absence of any commercial or financial relationships that could be construed as a potential conflict of interest.
