# Supplementary material for: Luteal Lipids Regulate Progesterone Production and May Modulate Immune Cell Function During the Estrous Cycle and Pregnancy
Source: Front Endocrinol (Lausanne). 2019 Oct 4;10:662. doi: 10.3389/fendo.2019.00662 (PMC6788218; doi:10.3389/fendo.2019.00662)
Supplement: Supplementary file 1 [file Data_Sheet_1.docx]

**Supplemental Table 1: OxyEndo UPLC Solvent Gradient**

| **Time** | **% B** |
| --- | --- |
| 0.01 | 25 |
| 1.00 | 40 |
| 2.50 | 42 |
| 4.50 | 50 |
| 10.50 | 65 |
| 12.50 | 75 |
| 13.25 | 80 |
| 17.25 | 85 |
| 18.25 | 95 |
| 18.75 | 100 |
| 19.00 | 100 |
| 19.10 | 25 |
| 20.00 | stop |

Solvent A: 0.1% Acetic Acid in Water

Solvent B: 10:90 Isopropanol:Acetonitrile

Flow: 250 µL/min

**Supplemental Table 2: Sciex QTRAP Global MS Parameters for Oxylipin and Endocannabinoid Assays**

| **Assay** | **Mode** | **IS**  **(V)** | **TEM (ºC)** | **CUR (L/min)** | **GS1 (L/min)** | **GS2 (L/min)** | **CAD (Pressure)** | |
| --- | --- | --- | --- | --- | --- | --- | --- | --- |
| Oxylipin | -ESI | -4500 | 525 | 35 | 60 | 50 | Med | |
| Endo | +ESI | 5500 | 525 | 35 | 60 | 50 | Med | |
| CUR - curtain gas flow; CAD - collision gas pressure setting; CAD - collision gas pressure setting; IS - ion source voltage; TEM - source temperature; GS1 - nebulizer gasGS2 - heater gas | | | | | | | |  |
|  |  |  |  |  |  |  |  |  |
|  |  |  |  |  |  |  |  |  |

**Supplemental Table 3: Oxy/Endo -ESI Analyte Specific Parameters for Sciex 6500 QTRAP**

| # | Analyte | tR | Q1 | Q3 | DCP | EP | CE | CXP | SSTD |
| --- | --- | --- | --- | --- | --- | --- | --- | --- | --- |
| 1 | PUHA -esi | 3.06 | 249 | 130 | -30 | -10 | -18 | -13 | --- |
| 2 | 6-keto PGF1α-d4 | 3.54 | 373 | 167 | -70 | -10 | -36 | -13 | 6-keto PGF1α-d4 |
| 3 | 6-keto PGF1α | 3.51 | 369 | 163 | -70 | -10 | -33 | -13 | 6-keto PGF1α-d4 |
| 4 | PGF3α | 4.12 | 351 | 307 | -60 | -10 | -24 | -13 | PGF2α-d4 |
| 5 | PGE3 | 4.25 | 349 | 269 | -35 | -10 | -21 | -13 | PGD2-d4 |
| 6 | TXB2-d4 | 4.55 | 373 | 173 | -50 | -10 | -45 | -13 | PUHA -esi |
| 7 | TXB2 | 4.61 | 369 | 169 | -50 | -10 | -21 | -13 | TXB2-d4 |
| 8 | 9_12_13-TriHOME | 4.82 | 329 | 211 | -60 | -10 | -30 | -13 | PGF2α-d4 |
| 9 | PGF2α-d4 | 4.87 | 357 | 197 | -70 | -10 | -33 | -13 | CUDA -esi |
| 10 | PGF2α | 4.89 | 353 | 193 | -70 | -10 | -33 | -13 | PGF2α-d4 |
| 11 | PGE2 | 4.99 | 351 | 271 | -40 | -10 | -21 | -13 | PGD2-d4 |
| 12 | PGE1 | 5.15 | 353 | 317 | -40 | -10 | -18 | -13 | PGD2-d4 |
| 13 | PGD2-d4 | 5.16 | 355 | 275 | -40 | -10 | -27 | -13 | CUDA -esi |
| 14 | PGD2 | 5.26 | 351 | 271 | -40 | -10 | -24 | -13 | PGD2-d4 |
| 15 | 15-Keto PGE2 | 5.3 | 349 | 331 | -50 | -10 | -15 | -13 | PGD2-d4 |
| 16 | Resolvin D1 | 5.78 | 375 | 121 | -60 | -10 | -39 | -13 | PGD2-d4 |
| 17 | Lipoxin A4 | 5.89 | 351 | 217 | -45 | -10 | -27 | -13 | PGD2-d4 |
| 18 | LTB5 | 6.99 | 333 | 195 | -50 | -10 | -21 | -13 | LTB4-d4 |
| 19 | 15_16-DiHODE | 7.49 | 311 | 235 | -55 | -10 | -27 | -13 | 14_15-DiHETrE-d11 |
| 20 | 8_15-DiHETE | 7.58 | 335 | 235 | -65 | -10 | -21 | -13 | 14_15-DiHETrE-d11 |
| 21 | 12_13-DiHODE | 7.59 | 311 | 183 | -60 | -10 | -27 | -13 | 14_15-DiHETrE-d11 |
| 22 | 9_10-DiHODE | 7.64 | 311 | 201 | -65 | -10 | -27 | -13 | 14_15-DiHETrE-d11 |
| 23 | 17_18-DiHETE | 7.8 | 335 | 247 | -70 | -10 | -24 | -13 | 14_15-DiHETrE-d11 |
| 24 | 5_15-DiHETE | 7.81 | 335 | 173 | -40 | -10 | -18 | -13 | 14_15-DiHETrE-d11 |
| 25 | 6-trans-LTB4 | 7.86 | 335 | 195 | -85 | -10 | -21 | -13 | LTB4-d4 |
| 26 | CUDA -esi | 8.7 | 339 | 214 | -70 | -10 | -30 | -13 | --- |
| 27 | LTB4-d4 | 8.48 | 339 | 163 | -70 | -10 | -33 | -13 | CUDA -esi |
| 28 | 14_15-DiHETE | 8.51 | 335 | 207 | -40 | -10 | -24 | -13 | 14_15-DiHETrE-d11 |
| 29 | LTB4 | 8.58 | 335 | 195 | -50 | -10 | -24 | -13 | LTB4-d4 |
| 30 | 12_13-DiHOME | 8.9 | 313 | 183 | -70 | -10 | -30 | -13 | 14_15-DiHETrE-d11 |
| 31 | 9_10-DiHOME | 9.34 | 313 | 201 | -65 | -10 | -27 | -13 | 14_15-DiHETrE-d11 |
| 32 | 14_15-DiHETrE-d11 | 9.5 | 348 | 207 | -65 | -10 | -24 | -13 | CUDA -esi |
| 33 | 19_20-DiHDoPE | 9.55 | 361 | 273 | -80 | -10 | -24 | -13 | 14_15-DiHETrE-d11 |
| 34 | 14_15-DiHETrE | 9.6 | 337 | 207 | -60 | -10 | -24 | -13 | 14_15-DiHETrE-d11 |
| 35 | 11_12-DiHETrE | 10.2 | 337 | 167 | -55 | -10 | -27 | -13 | 14_15-DiHETrE-d11 |
| 36 | 9_10-e-DiHO | 10.4 | 315 | 297 | -110 | -10 | -30 | -13 | 14_15-DiHETrE-d11 |

CUR - curtain gas flow; CAD - collision gas pressure setting; CAD - collision gas pressure setting; IS - ion source voltage; TEM - source temperature; GS1 - nebulizer gasGS2 - heater gas

**Supplemental Table 3 (Cont): Oxy/Endo –ESI Analyte Specific Parameters for Sciex 6500 QTRAP**

| # | Analyte | tR | Q1 | Q3 | DCP | EP | CE | CXP | SSTD |
| --- | --- | --- | --- | --- | --- | --- | --- | --- | --- |
| 37 | 12(13)-Ep-9-KODE | 10.4 | 309 | 291 | -90 | -10 | -21 | -13 | 9-HODE-d4 |
| 38 | 13-HOTrE | 10.6 | 293 | 195 | -70 | -10 | -24 | -13 | 9-HODE-d4 |
| 39 | 8_9-DiHETrE | 10.8 | 337 | 127 | -65 | -10 | -27 | -13 | 14_15-DiHETrE-d11 |
| 40 | 15-deoxy PGJ2 | 10.8 | 315 | 271 | -50 | -10 | -21 | -13 | PGD2-d4 |
| 41 | 20-HETE-d6 | 11 | 325 | 281 | -80 | -10 | -21 | -13 | CUDA -esi |
| 42 | 15-HEPE | 11 | 317 | 219 | -55 | -10 | -18 | -13 | 12-HETE-d8 |
| 43 | 20-HETE | 11 | 319 | 275 | -95 | -10 | -24 | -13 | 20-HETE-d6 |
| 44 | 12-HEPE | 11.4 | 317 | 179 | -45 | -10 | -18 | -13 | 12-HETE-d8 |
| 45 | 5_6-DiHETrE | 11.5 | 337 | 145 | -55 | -10 | -24 | -13 | 14_15-DiHETrE-d11 |
| 46 | 9-HEPE | 11.6 | 317 | 167 | -45 | -10 | -18 | -13 | 12-HETE-d8 |
| 47 | 13-HODE | 11.9 | 295 | 195 | -90 | -10 | -24 | -13 | 9-HODE-d4 |
| 48 | 5-HEPE | 12 | 317 | 115 | -40 | -10 | -21 | -13 | 5—HETE-d8 |
| 49 | 9-HODE-d4 | 12 | 299 | 172 | -85 | -10 | -27 | -13 | CUDA -esi |
| 50 | 9-HODE | 12.1 | 295 | 171 | -70 | -10 | -24 | -13 | 9-HODE-d4 |
| 51 | 15(16)-EpODE | 12.2 | 293 | 275 | -75 | -10 | -18 | -13 | 12(13)-EpOME-d4 |
| 52 | 17(18)-EpETE | 12.3 | 317 | 259 | -55 | -10 | -15 | -13 | 12(13)-EpOME-d4 |
| 53 | 15-HETE | 12.3 | 319 | 219 | -55 | -10 | -18 | -13 | 12-HETE-d8 |
| 54 | 13-KODE | 12.3 | 293 | 179 | -80 | -10 | -27 | -13 | 9-HODE-d4 |
| 55 | 9(10)-EpODE | 12.4 | 293 | 275 | -65 | -10 | -18 | -13 | 12(13)-EpOME-d4 |
| 56 | 9-HOTrE | 10.4 | 293 | 171 | -60 | -10 | -21 | -13 | 9-HODE-d4 |
| 57 | 17-HDoHE | 12.4 | 343 | 281 | -55 | -10 | -18 | -13 | 12-HETE-d8 |
| 58 | 12(13)-EpODE | 12.5 | 293 | 183 | -50 | -10 | -24 | -13 | 12(13)-EpOME-d4 |
| 59 | 15-KETE | 12.6 | 317 | 273 | -60 | -10 | -18 | -13 | 12-HETE-d8 |
| 60 | 14-HDoHE | 12.7 | 343 | 281 | -60 | -10 | -18 | -13 | 12-HETE-d8 |
| 61 | 11-HETE | 12.7 | 319 | 167 | -45 | -10 | -21 | -13 | 12-HETE-d8 |
| 62 | 14(15)-EpETE | 12.7 | 317 | 247 | -35 | -10 | -18 | -13 | 12(13)-EpOME-d4 |
| 63 | 9-KODE | 12.8 | 293 | 185 | -100 | -10 | -27 | -13 | 9-HODE-d4 |
| 64 | 12-HETE-d8 | 12.8 | 327 | 184 | -60 | -10 | -21 | -13 | CUDA -esi |
| 65 | 11(12)-EpETE | 12.8 | 317 | 167 | -40 | -10 | -18 | -13 | 12(13)-EpOME-d4 |
| 66 | 12-HETE | 12.9 | 319 | 179 | -60 | -10 | -21 | -13 | 12-HETE-d8 |
| 67 | 8-HETE | 13.1 | 319 | 155 | -45 | -10 | -21 | -13 | 12-HETE-d8 |
| 68 | 9-HETE | 13.2 | 319 | 167 | -60 | -10 | -18 | -13 | 12-HETE-d8 |
| 69 | 5-HETE-d8 | 13.5 | 327 | 116 | -70 | -10 | -18 | -13 | CUDA -esi |
| 70 | 19(20)-EpDoPE | 13.5 | 343 | 281 | -60 | -10 | -18 | -13 | 12(13)-EpOME-d4 |
| 71 | 5-HETE | 13.5 | 319 | 115 | -50 | -10 | -18 | -13 | 5-HETE-d8 |
| 72 | 12(13)-EpOME-d4 | 13.6 | 299 | 198 | -90 | -10 | -24 | -13 | CUDA -esi |

CUR - curtain gas flow; CAD - collision gas pressure setting; CAD - collision gas pressure setting; IS - ion source voltage; TEM - source temperature; GS1 - nebulizer gasGS2 - heater gas

**Supplemental Table 3: Oxy/Endo –ESI Analyte Specific Parameters for Sciex 6500 QTRAP**

| # | Analyte | tR | Q1 | Q3 | DCP | EP | CE | CXP | SSTD |
| --- | --- | --- | --- | --- | --- | --- | --- | --- | --- |
| 73 | 12(13)-EpOME | 13.7 | 295 | 195 | -85 | -10 | -21 | -13 | 12(13)-EpOME-d4 |
| 74 | 14(15)-EpETrE | 13.8 | 319 | 219 | -50 | -10 | -18 | -13 | 12(13)-EpOME-d4 |
| 75 | 4-HDoHE | 13.8 | 343 | 281 | -60 | -10 | -18 | -13 | 12-HETE-d8 |
| 76 | 16(17)-EpDoPE | 13.9 | 343 | 274 | -45 | -10 | -15 | -13 | 12(13)-EpOME-d4 |
| 77 | 9(10)-EpOME | 13.9 | 295 | 171 | -75 | -10 | -21 | -13 | 12(13)-EpOME-d4 |
| 78 | 5-KETE | 14.2 | 317 | 203 | -75 | -10 | -27 | -13 | 5-HETE-d8 |
| 79 | 11(12)-EpETrE | 14.2 | 319 | 167 | -40 | -10 | -21 | -13 | 12(13)-EpOME-d4 |
| 80 | 8(9)-EpETrE | 14.4 | 319 | 155 | -40 | -10 | -18 | -13 | 12(13)-EpOME-d4 |
| 81 | 10-Nitrolinoleate | 14.6 | 324 | 277 | -40 | -10 | -18 | -13 | 10-Nitrooleate-d17 |
| 82 | 9_10-EpO | 15.1 | 297 | 279 | -105 | -10 | -27 | -13 | 12(13)-EpOME-d4 |
| 83 | 10-Nitrooleate-d17 | 15.3 | 343 | 308 | -65 | -10 | -18 | -13 | CUDA -esi |
| 84 | 10-Nitrooleate | 15.4 | 326 | 280 | -40 | -10 | -24 | -13 | 10-Nitrooleate-d17 |
| 85 | 9-Nitrooleate | 15.5 | 326 | 308 | -50 | -10 | -18 | -13 | 10-Nitrooleate-d17 |
| 86 | C20:5n3 | 15.5 | 301 | 257 | -60 | -10 | -15 | -13 | C20:4n6-d8 |
| 87 | C18:3n3 | 15.6 | 277 | 259 | -115 | -10 | -24 | -13 | C20:4n6-d8 |
| 88 | C22:6n3 | 16.2 | 327 | 283 | -45 | -10 | -15 | -13 | C20:4n6-d8 |
| 89 | C20:4n6-d8 | 16.5 | 311 | 267 | -60 | -10 | -18 | -13 | C20:4n6-d8 |
| 90 | C20:4n6 | 16.6 | 303 | 259 | -40 | -10 | -18 | -13 | C20:4n6-d8 |
| 91 | C18:2n6 | 16.9 | 279 | 261 | -185 | -10 | -38 | -13 | C20:4n6-d8 |

CUR - curtain gas flow; CAD - collision gas pressure setting; CAD - collision gas pressure setting; IS - ion source voltage; TEM - source temperature; GS1 - nebulizer gasGS2 - heater gas

**Supplemental Table 4: Oxy/Endo +ESI Analyte Specific Parameters for Sciex 6500 QTRAP**

| # | Analyte | tR | Q1 | Q3 | DCP | EP | CE | CXP | SSTD |
| --- | --- | --- | --- | --- | --- | --- | --- | --- | --- |
| 1 | PUHA +esi | 3.06 | 251.2 | 114.1 | 65 | 10 | 21 | 12 | --- |
| 2 | PGF2α-EA-d4 | 3.62 | 384.3 | 62.1 | 50 | 10 | 42 | 12 | PUHA +esi |
| 3 | PGF2a-EA | 3.63 | 380.3 | 62.1 | 45 | 10 | 39 | 12 | PGF2α-EA-d4 |
| 4 | PGE2-EA | 3.65 | 378.301 | 62.1 | 65 | 10 | 39 | 12 | PGF2α-EA-d4 |
| 5 | PGD2-EA | 3.92 | 378.302 | 62.1 | 65 | 10 | 42 | 12 | PGF2α-EA-d4 |
| 6 | PGF2a 1G | 4.29 | 411.3 | 301.2 | 40 | 10 | 21 | 12 | PGF2α-EA-d4 |
| 7 | PGE2 1G | 4.33 | 409.3 | 317.2 | 75 | 10 | 21 | 12 | PGF2α-EA-d4 |
| 8 | CUDA +esi | 8.7 | 341.3 | 216.2 | 50 | 10 | 24 | 12 | --- |
| 9 | 15-HETE-EA | 9.62 | 346.3 | 62.1 | 75 | 10 | 21 | 12 | A-EA-d8 |
| 10 | 11(12)-EpETre-EA | 11.73 | 364.3 | 62.1 | 75 | 10 | 45 | 12 | A-EA-d8 |
| 11 | aL-EA | 13.46 | 322.2 | 62.1 | 60 | 10 | 21 | 12 | A-EA-d8 |
| 12 | DH-EA | 14.61 | 372.3 | 62.1 | 55 | 10 | 45 | 12 | A-EA-d8 |
| 13 | A-EA-d8 | 14.72 | 356.3 | 63.1 | 50 | 10 | 45 | 12 | CUDA +esi |
| 14 | A-EA | 14.78 | 348.3 | 62.1 | 70 | 10 | 39 | 12 | A-EA-d8 |
| 15 | L-EA | 14.81 | 324.2 | 62.1 | 70 | 10 | 21 | 12 | A-EA-d8 |
| 16 | NA-Gly-d8 | 14.86 | 370.3 | 76.1 | 45 | 10 | 21 | 12 | CUDA +esi |
| 17 | NA-Gly | 14.91 | 362.3 | 76.1 | 65 | 10 | 21 | 12 | NA-Gly-d8 |
| 18 | 2-AG-d5 | 15.41 | 384.3 | 287.2 | 60 | 10 | 21 | 12 | CUDA +esi |
| 19 | 2-AG | 15.43 | 379.3 | 287.2 | 110 | 10 | 24 | 12 | 2-AG-d5 |
| 20 | DGL- EA | 15.45 | 350.3 | 62.1 | 35 | 10 | 36 | 12 | A-EA-d8 |
| 21 | 2-LG | 15.57 | 355.3 | 263.2 | 25 | 10 | 12 | 12 | 2-AG-d5 |
| 22 | P-EA-d4 | 15.62 | 304.2 | 62.1 | 90 | 10 | 18 | 12 | CUDA +esi |
| 23 | 1-AG | 15.64 | 379.3 | 287.2 | 95 | 10 | 21 | 12 | 2-AG-d5 |
| 24 | P-EA | 15.64 | 300.2 | 62.1 | 70 | 10 | 18 | 12 | P-EA-d4 |
| 25 | 1-LG | 15.82 | 355.3 | 263.2 | 40 | 10 | 12 | 12 | 2-AG-d5 |
| 26 | O-EA | 16.1 | 326.2 | 62.1 | 105 | 10 | 21 | 12 | A-EA-d8 |
| 27 | D-EA | 16.12 | 376.3 | 62.1 | 105 | 10 | 45 | 12 | A-EA-d8 |
| 28 | NO-Gly | 16 | 340.2 | 76.2 | 60 | 10 | 21 | 12 | NA-Gly-d8 |
| 29 | 2-OG | 16.97 | 357.3 | 265.2 | 50 | 10 | 15 | 12 | 2-AG-d5 |
| 30 | 1-OG | 17.31 | 357.3 | 265.2 | 55 | 10 | 15 | 12 | 2-AG-d5 |
| 31 | S-EA | 17.79 | 328.2 | 62.1 | 100 | 10 | 21 | 12 | P-EA-d4 |

CUR - curtain gas flow; CAD - collision gas pressure setting; CAD - collision gas pressure setting; IS - ion source voltage; TEM - source temperature; GS1 - nebulizer gasGS2 - heater gas
