# Supplementary figures and images for: Luteal Lipids Regulate Progesterone Production and May Modulate Immune Cell Function During the Estrous Cycle and Pregnancy
Source: Front Endocrinol (Lausanne). 2019 Oct 4;10:662. doi: 10.3389/fendo.2019.00662 (PMC6788218; doi:10.3389/fendo.2019.00662)

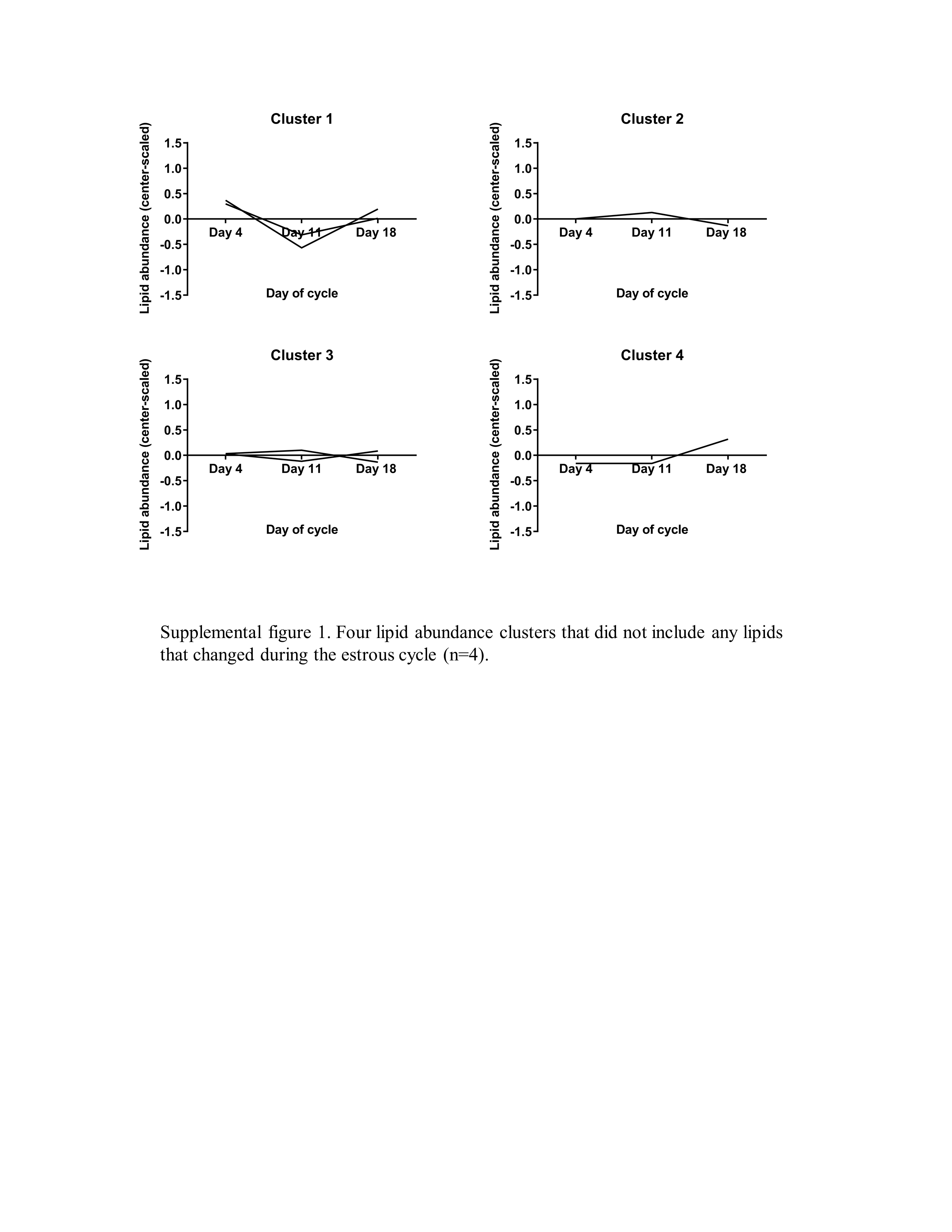

Supplement: Supplementary file 3 [file Image_1.TIF]
